# MiR‐552 promotes the proliferation, migration and EMT of hepatocellular carcinoma cells by inhibiting *AJAP1* expression

**DOI:** 10.1111/jcmm.14062

**Published:** 2018-12-30

**Authors:** Weiqing Qu, Xinyuan Wen, Keli Su, Wei Gou

**Affiliations:** ^1^ Department of Oncology Yantaishan Hospital Yantai, Shandong China; ^2^ Affiliated Hospital of Jining Medical University Jining, Shandong China; ^3^ Department of Oncology Fourth People's Hospital of Jinan Jinan, Shandong China; ^4^ The Sixth Department of Hepatopathy Qingdao No. 6 People's Hospital Qingdao, Shandong China

**Keywords:** AJAP1, EMT, hepatocellular carcinoma, miR‐552

## Abstract

Our goal was to explore the function of miR‐552 and its potential target *AJAP1* in hepatocellular carcinoma (HCC) oncogenesis and progression. In this study, bioinformatics analysis was performed to detect abnormally expressed miRNAs. The relationship between miR‐552 and *AJAP1* was validated using luciferase reporter assays. RT‐qPCR and Western blot assays were applied to explore the expression level of miR‐552, *AJAP1* and epithelial‐mesenchymal transition (EMT) markers. HCC cell proliferation was examined using CCK8 assays, while migration and invasion were investigated using Transwell assays. Nude mouse tumourigenesis models were established to facilitate observation of HCC progression in vivo. Finally, prognostic analysis was performed to discover how the prognosis of HCC patients correlated with miR‐552 and *AJAP1* expression. MiR‐552 overexpression in HCC cells promoted HCC cell migration, invasion and EMT by targeting/suppressing *AJAP1*. Poorer prognosis appeared in HCC patients with higher miR‐552 expression or lower *AJAP1* levels. Our findings suggested that miR‐552 promotes HCC oncogenesis and progression by inhibiting *AJAP1* expression.

## 1 INTRODUCTION

Hepatocellular carcinoma (HCC) ranks as the fifth most prevalent malignancy worldwide.^1^ As it is the most frequent primary liver cancer, HCC is the second most deadly cancer, with a 5‐year survival rate of <20%.^2^, ^3^ Patients with chronic liver disease are at high risk for HCC oncogenesis.^4^ However, because early‐stage HCC is often not symptomatic, it is not easily identified; this feature, in combination with its common recurrence and metastasis after absolute resection, leads to poor recovery.^4^, ^5^ It timely to investigate the underlying pathogenic mechanisms of HCC to develop possible novel curative treatment methods.^1^

MicroRNAs (miRNAs) are sequences of small noncoding RNAs of 18‐22 nt in length.^6^ They manipulate the expression levels of more than 60% of human genes at the posttranscriptional level by binding to the 3′ untranslated regions (3′UTR) of their target messenger RNAs (mRNAs).^7^ This repression enables them to participate in cellular events, including proliferation, apoptosis and movement.^8^ The first attempt to elucidate how miRNAs participate in human cancers was made by Chen et al^9^ as early as in 2002. Recent studies have confirmed the involvement of miRNAs in HCC. For instance, Jiang et al^10^ reported significant downregulation of miR‐874 in HCC cells and tissues associated with clinical stage, and showed that miR‐847 overexpression inhibited tumour development. Li et al^11^, however, found miR‐155 upregulation and a promotive role in HCC cell invasion and migration. A number of studies have supported the conclusion that miR‐552 is possibly involved in the molecular mechanism of another cancer within the human digestive system. Cao et al^12^ concluded that upregulation of miR‐552 promoted colorectal cancer (CRC) cell growth by targeting *DACH1*. Wang et al. also found that *ADAM28* is a target of miR‐552 in CRC.^6^ In addition, miR‐552 was identified by Leivonen et al^7^ as a negative regulator of *HER2* in breast cancer. However, much remains unknown regarding the roles of miR‐552 in the regulation of HCC oncogenesis and progression.

Adherens junctions‐associated protein‐1 (*AJAP1*) is located on 1p36, a chromosome in which nonrandom deletion is often detected in various human malignancies.^13^ *AJAP1* has been widely acknowledged as a biomarker for glioblastoma (GBM), for example, by Yang et al^14^ Zeng et al^13^ argued for its positive correlation with poorer GBM survival. In addition, *AJAP1* could suppress cell adhesion and migration in oligodendrogliomas.^15^ In HCC cell lines and tissues, *AJAP1* loss was observed by Ezaka et al^16^, who highlighted not only its HCC‐suppressive role but also its intermediate role in the epithelial‐mesenchymal transition (EMT) process. As cell migration and invasion are the primary components of EMT, we therefore adopted EMT as a notable criterion.^14^ There is less documentation on how *AJAP1* is involved in HCC compared with other human cancers. Our study pioneered the exploration of the function of *AJAP1* in HCC development.

In the present study, bioinformatics analysis was conducted to identify differentially expressed miRNAs in HCC. The miR‐552 and *AJAP1* expression levels in HCC cells and tissues were determined. The expression levels of EMT markers were measured to confirm the influence of miR‐552/*AJAP1* on EMT. CCK8 and Transwell assays were used to study the regulatory effects of the *AJAP1* and miR‐552 interaction on HCC cell proliferation, migration and invasion. In addition to the prognostic analysis, an experiment using nude mice investigated this effect in vivo. Our research may open up a new path towards HCC treatment.

## 2 MATERIALS AND METHODS

### 2.1 Cell culture

Eighty‐one pairs of human HCC tissues and the corresponding adjacent tissues were obtained from Qingdao No. 6 People's Hospital. The 81 patients had undergone neither chemotherapy nor radiotherapy before the absolute resection. The detailed clinicopathological characteristics of these patients are provided in Table 1. The clinical HCC stages were based on the tumour‐node‐metastasis (TNM) Classification of Malignant Tumours by the Union for International Cancer Control (UICC). We received informed patient consents in written form, along with official approval by the Ethics Committee of Qingdao No. 6 People's Hospital. The Hep3B and HepG2 HCC cell lines were acquired from the American Type Culture Collection (ATCC, Manassas, VA, USA), while the Bel‐7404 and SMMC‐7721 cell lines were acquired from the BeNa Culture Collection (Beijing, China). The former two lines were cultivated in Dulbecco's modified Eagle's medium (Gibco, Grand Island, NY, USA) with 10% foetal bovine serum (FBS) at 37°C in a 5% CO_2_ atmosphere. The latter two lines were cultured in 90%RPMI‐1640 + 10%FBS. The L02 cell line was obtained from the Institute of Biochemistry and Cell Biology, Chinese Academy of Sciences (Shanghai, China).

**Table 1:** Correlation between miR‐552 level and clinicopathological characteristic of HCC patients

Feature	Cases	miR‐552 expression		*P*‐value
		Low (n = 35)	High (n = 46)	
Sex				
Male	68	30	38	
Female	13	5	8	0.706
Age				
>50	45	20	25	
≤50	36	15	21	0.802
AFT(ng/mL)				
>400	39	16	23	
≤400	42	20	22	0.551
Tumor size(cm)				
>5	51	19	32	
≤5	30	16	14	0.158
Tumor number				
Single	41	17	24	
Multiple	40	18	22	0.748
Histological grade				
G1	25	18	7	
G2 + G3	56	17	39	0.001**
TNM stage				
I + II	50	26	24	
III + IV	31	9	22	0.043*

Correlation between clinicopathological characteristics and miR‐552 expression was detected by chi‐square test. *P* < 0.05 was considered as significant. *indicated that *P* < 0.05 compared to I + II TNM stage, ^**^indicated that *P* < 0.01 compared to G1 grade tumor.

### 2.2 Bioinformatics analysis

Hepatocellular carcinoma miRNA chip analysis primary data were accessed at The Cancer Genome Atlas (TCGA) public database (http://www.cancergenome.nih.gov/dataportal). The HCC miRNA expression data were downloaded using the Genomic Data Commons (GDC) Data Transfer Tool on GDC DATA PORTAL (https://gdc-portal.nci.nih.gov/). The TCGA ID is listed in Table 2. Using R Project, miRNA expression data from in total 42 pairs of tumour and the matched adjacent tissues were analysed. The data were normalized using the DESeq2 package (http://bioconductor.org/biocLite.R). The significance levels of the difference between the mean expression values of the two groups were determined by Student's *t* test (unpaired, two‐tailed). A fold change |log (FC)|>2 and *P* < 0.05 were set as cut‐off criteria. The volcano plot and heat map of miRNAs were drawn according to the miRNA differential expression using the pheatmap package. The Kaplan‐Meier (KM) plots for the clinical follow‐up data in the TCGA database and the survival rates were executed using the survival package and survplot package based on R project.

**Table 2:** Sample ID from TCGA

Group	Sample ID	Group	Sample ID
Normal	TCGA‐DD‐A3A6‐11A‐11R‐A22J‐13	Tumour	TCGA‐DD‐A3A6‐01A‐11R‐A22J‐13
	TCGA‐DD‐A3A5‐11A‐11R‐A22J‐13		TCGA‐DD‐A3A5‐01A‐11R‐A22J‐13
	TCGA‐DD‐A3A4‐11A‐11R‐A22J‐13		TCGA‐DD‐A3A4‐01A‐11R‐A22J‐13
	TCGA‐DD‐A3A3‐11A‐11R‐A22J‐13		TCGA‐DD‐A3A3‐01A‐11R‐A22J‐13
	TCGA‐DD‐A3A2‐11A‐11R‐A214‐13		TCGA‐DD‐A3A2‐01A‐11R‐A214‐13
	TCGA‐DD‐A3A1‐11A‐11R‐A214‐13		TCGA‐DD‐A3A1‐01A‐11R‐A214‐13
	TCGA‐DD‐A3A8‐11A‐11R‐A22J‐13		TCGA‐DD‐A3A8‐01A‐11R‐A22J‐13
	TCGA‐BD‐A3EP‐11A‐12R‐A22J‐13		TCGA‐BD‐A3EP‐01A‐11R‐A22J‐13
	TCGA‐DD‐A11D‐11A‐12R‐A130‐13		TCGA‐DD‐A11D‐01A‐11R‐A130‐13
	TCGA‐FV‐A23B‐11A‐11R‐A16S‐13		TCGA‐FV‐A23B‐01A‐11R‐A16S‐13
	TCGA‐DD‐A1EL‐11A‐11R‐A154‐13		TCGA‐DD‐A1EL‐01A‐11R‐A154‐13
	TCGA‐DD‐A1EI‐11A‐11R‐A130‐13		TCGA‐DD‐A1EI‐01A‐11R‐A130‐13
	TCGA‐DD‐A1EH‐11A‐11R‐A130‐13		TCGA‐DD‐A1EH‐01A‐11R‐A130‐13
	TCGA‐DD‐A1EJ‐11A‐11R‐A154‐13		TCGA‐DD‐A1EJ‐01A‐11R‐A154‐13
	TCGA‐DD‐A1EE‐11A‐11R‐A130‐13		TCGA‐DD‐A1EE‐01A‐11R‐A130‐13
	TCGA‐DD‐A1EG‐11A‐11R‐A214‐13		TCGA‐DD‐A1EG‐01A‐11R‐A214‐13
	TCGA‐FV‐A3I1‐11A‐11R‐A22J‐13		TCGA‐FV‐A3I1‐01A‐11R‐A22J‐13
	TCGA‐DD‐A1EB‐11A‐11R‐A130‐13		TCGA‐DD‐A1EB‐01A‐11R‐A130‐13
	TCGA‐EP‐A26S‐11A‐12R‐A16S‐13		TCGA‐EP‐A26S‐01A‐11R‐A16S‐13
	TCGA‐FV‐A2QR‐11A‐11R‐A214‐13		TCGA‐FV‐A2QR‐01A‐11R‐A214‐13
	TCGA‐BC‐A10W‐11A‐11R‐A130‐13		TCGA‐BC‐A10W‐01A‐11R‐A130‐13
	TCGA‐BC‐A10T‐11A‐11R‐A130‐13		TCGA‐BC‐A10T‐01A‐11R‐A130‐13
	TCGA‐BC‐A10U‐11A‐11R‐A130‐13		TCGA‐BC‐A10U‐01A‐11R‐A130‐13
	TCGA‐BC‐A10R‐11A‐11R‐A130‐13		TCGA‐BC‐A10R‐01A‐11R‐A130‐13
	TCGA‐BC‐A10Q‐11A‐11R‐A130‐13		TCGA‐BC‐A10Q‐01A‐11R‐A130‐13
	TCGA‐BC‐A10Z‐11A‐11R‐A130‐13		TCGA‐BC‐A10Z‐01A‐11R‐A130‐13
	TCGA‐BC‐A10X‐11A‐11R‐A130‐13		TCGA‐BC‐A10X‐01A‐11R‐A130‐13
	TCGA‐BC‐A10Y‐11A‐11R‐A130‐13		TCGA‐BC‐A10Y‐01A‐11R‐A130‐13
	TCGA‐ES‐A2HT‐11A‐11R‐A17X‐13		TCGA‐ES‐A2HT‐01A‐12R‐A17X‐13
	TCGA‐BC‐A216‐11A‐11R‐A154‐13		TCGA‐BC‐A216‐01A‐11R‐A154‐13
	TCGA‐G3‐A3CH‐11A‐11R‐A22J‐13		TCGA‐G3‐A3CH‐01A‐11R‐A22J‐13
	TCGA‐BD‐A2L6‐11A‐21R‐A214‐13		TCGA‐BD‐A2L6‐01A‐11R‐A214‐13
	TCGA‐DD‐A39Z‐11A‐21R‐A214‐13		TCGA‐DD‐A39Z‐01A‐11R‐A214‐13
	TCGA‐DD‐A39X‐11A‐11R‐A214‐13		TCGA‐DD‐A39X‐01A‐11R‐A214‐13
	TCGA‐DD‐A39W‐11A‐11R‐A214‐13		TCGA‐DD‐A39W‐01A‐11R‐A214‐13
	TCGA‐DD‐A39V‐11A‐11R‐A214‐13		TCGA‐DD‐A39V‐01A‐11R‐A214‐13
	TCGA‐EP‐A3RK‐11A‐11R‐A22J‐13		TCGA‐EP‐A3RK‐01A‐11R‐A22J‐13
	TCGA‐BC‐A110‐11A‐11R‐A130‐13		TCGA‐BC‐A110‐01A‐11R‐A130‐13
	TCGA‐DD‐A119‐11A‐11R‐A130‐13		TCGA‐DD‐A119‐01A‐11R‐A130‐13
	TCGA‐DD‐A118‐11A‐11R‐A130‐13		TCGA‐DD‐A118‐01A‐11R‐A130‐13
	TCGA‐DD‐A11A‐11A‐11R‐A130‐13		TCGA‐DD‐A11A‐01A‐11R‐A130‐13
	TCGA‐DD‐A11C‐11A‐11R‐A130‐13		TCGA‐DD‐A11C‐01A‐11R‐A130‐13

### 2.3 Reverse transcription quantitative real‐time PCR

Total RNA was extracted from the cultured cell lines and tissues using Trizol Reagent (Invitrogen, Carlsbad, CA, USA). At room temperature, cells were lysed in Eppendorf tubes with Trizol and incubated for 10 minutes. Soybean‐sized HCC or normal tissues were ground in liquid nitrogen (LN) and incubated in the same environment after homogenisation. Precipitation of RNA in the colourless upper aqueous phase was performed with isopropyl alcohol. Next, the RNA samples were washed and precipitated with 75% ethanol. Finally, the air‐dried RNA pellets were dissolved in diethylpyrocarbonate and kept at −20°C for reverse transcription quantitative real‐time PCR (RT‐qPCR). The RNA concentrations were measured using an ultraviolet spectrophotometer.

The reverse transcription reaction was carried out by incubating the extracted RNA in a water bath for 1 hour at 37°C to synthesize cDNA, which was then used as a template in PCR reactions for amplification. Agarose gel electrophoresis was used to analyse the PCR products. The primer sequences used in the RT‐qPCR (Invitrogen) are listed in Table 3. GAPDH was used as an internal control for calculating the PRM1 mRNA content. Meanwhile, PRM1 standards with concentrations of 100, 10, 1, 0.1 and 0.01 ng, along with a negative contrast group were prepared. The expression levels of PRM1 mRNA were calculated after the plotting of a PCR reaction standard curve. The results were analysed using an Applied Biosystems 7300 Fast Real‐Time PCR System.

**Table 3:** Primer sequences for RT‐qPCR

Gene	Sequence (5′‐3′)
AJAP1 (HS1706206078)	
Forward	TCTGAGGCCCCGCTCCCCGAAACGTGA
Reverse	GGCGTCTGCCCTGCCCCCAGGAGGTAAA
GAPDH (HS1706206080)	
Forward	AAATGGTGAAGGTCGGTGTGAAC
Reverse	CAACAATCTCCACTTTGCCACTG
MiR‐552 (HS1706216091)	
Forward	CCGCACAGGTGACTGGTTAGA
Reverse	GTGCAGGGTCCGAGGT
U6 (HS1706216092)	
Forward	CTCGCTTCGGCAGCACATA
Reverse	AACGATTCACGAATTTGCGT

### 2.4 Western blotting

Normal hepatic or HCC tissues were ground in LN and mixed with lysis buffer for ultrasonication and centrifugation. The total protein concentrations of the supernatants were measured using the BCA Protein Assay Kit (Pierce Biotechnology, Rockford, IL, USA), and discontinuous sodium dodecyl sulphate polyacrylamide gel electrophoresis with different gel concentrations, depending on the relevant proteins’ molecular weights were used. After electrophoresis, the samples were transferred onto nitrocellulose (NC) membranes. The membranes were soaked in buffer containing 5% skimmed milk powder for 1 hour. The blots were incubated with primary antibodies, including anti‐AJAP1 (ab223117, 1:500; Abcam, Cambridge, MA, USA), anti‐E‐cadherin (ab1416, 1:500), anti‐Vimentin (ab92547, 1:500), anti‐N‐cadherin (ab213756, 1:500) and anti‐ZO‐1 (ab59720, 1:500), at 4°C overnight. Then, the membranes were the washed with Tris‐buffered saline Tween buffer and incubated at room temperature with HRP‐rabbit (#8114, 1:5000; Cell Signaling Technology, Danvers, MA, USA) and HRP‐mouse (#8125; 1:5000) for 1 hour. An Odyssey Infrared Imaging System was used to scan the membranes for further analysis. GAPDH (1:5000, HRP‐60004; Proteintech Group, Chicago, IL, USA) was used as a loading control. Band intensities were quantified using ImageJ software. The obtained images were converted into 8‐bit format to perform uncalibrated optical densitometry analysis. After conversion, the films were compared with the densitometry quantification setting the same ball radius value (50.0 pixels) for background subtraction. Each band was individually selected and circumscribed with the rectangular ROI selection tool and “Gels” function followed by quantification of the peak area in the obtained histograms. The data were acquired as arbitrary area values.

### 2.5 CCK8 assay

A CCK8 Kit (Dojindo, Shanghai, China) was used for the CCK8 assays. A defined number of cells were centrifuged after collection, resuspended with fresh solution and then seeded into 96‐well plates at a density of 200 μL or 1 × 10^4^ cells per well. The plates were incubated at 37°C for 1.5 hour. The absorbance values at OD 490 nm were measured each day using an ELISA plate reader (Biotek, Winooski, VT, USA).

### 2.6 Cell transfection

The miRNA mimics were synthesized via chemical synthesis to enhance the function of the endogenous miRNAs. The miRNA inhibitors are chemically modified suppressors that target specific miRNAs in the cells. The negative controls (NC) were scrambled oligonucleotides. Cell transfection was performed as described previously.^17^, ^18^ Briefly, the miR‐552 mimics, inhibitors and NC purchased from RiboBio (Guangzhou, China) were transfected into cells at a concentration of 100 nmol/L via Lipofectamine 3000 (Invitrogen) according to the manufacturer's protocol. The same approach was used for empty plasmids and *AJAP1* plasmids (1 μg; Origene, Rockville, MD, USA). The cells were transfected twice within 48 hours for the follow‐up experiments. The transfection efficiency was tested using RT‐qPCR.

### 2.7 Transwell migration assay

To perform the cell migration assay, 200 μL of cell suspension (1 × 10^5^ cells) was placed into the upper compartment of a Transwell chamber (Corning, Corning, NY, USA) with an 8 μm pore size with a 24‐well insert. In each well, 50 μL of serum‐free medium with 10 g/L bovine serum albumin was mixed with the HCC cells in the upper chamber. All the lower chambers, contained 10% FBS. The number of cells reaching the lower chamber shows migration ability.

### 2.8 Transwell invasion assay

Chambers were wrapped with Matrigel (BD Biosciences, San Jose, CA, USA) in the upper chamber for invasion assays. Serum‐free cell suspensions were seeded to the upper chambers and 10% FBS was added to the lower chambers. Crystal violet (0.1%) was used to stain the bottom cells on the membrane, after which the cells on the bottom of the chambers were imaged with a microscope. The number of cells in the lower chambers shows the invasion ability.

### 2.9 Luciferase reporter assay

**Figure 1:**
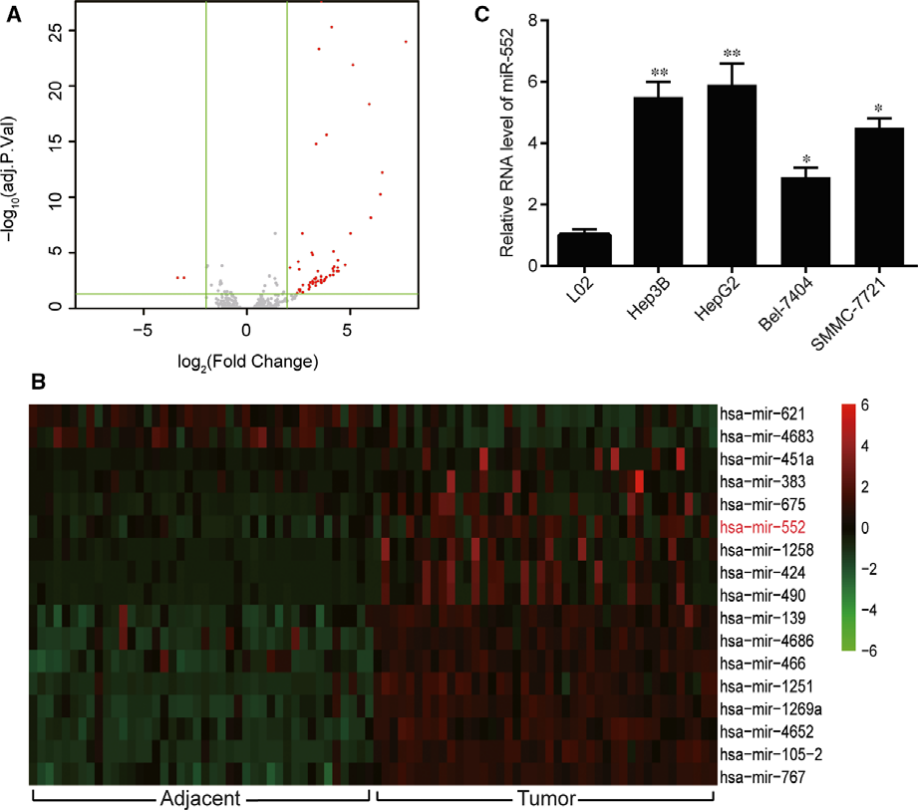
MiR‐552 was overexpressed in hepatocellular carcinoma (HCC) tissues and cell lines. (A) The volcano plot used a fold change (FC) value higher than 2 (|logFC|>2) and *P *<* *0.05 as cut‐off criteria for differentially expressed mRNAs. 17 miRNAs were marked as significantly expressed. (B) miR‐552 was among the 17 overexpressed mRNAs. (C) The RT‐qPCR result showed that the relative miR‐552 expression levels in HCC cells, including Hep3B, SMMC‐7721, Bel‐7404 and HepG2 were significantly higher than those in normal cells. The miR‐552 level was the highest in the Hep3B and HepG2 cell lines. *, *P *<* *0.05, **, *P *<* *0.01 compared to the L02 (normal) group

The target gene *AJAP1* was identified via TargetScan before investigating the correlation between *AJAP1* and miR‐552 using luciferase reporter assays. Wild‐type (WT) and mutated (MUT) versions of the *AJAP1* 3′‐UTR were inserted into pGL3 plasmids (Promega, Madison, WI, USA). MiR‐552 mimics (Sangon, Shanghai, China) or control mimics, as well as pGL3 plasmids, were cotransfected using Lipofectamine 3000 into Hep3B and HepG2 cells. Details for the sequences of the WT and MUT 3′‐UTRs are given in Figure 1A. Renilla (Promega) activity was used as an internal control. Two days (48 h) after transfection, the relative luciferase activity levels were analysed using a Dual‐Luciferase Reporter Assay System (Promega).

### 2.10 Nude mice subcutaneous tumour model establishment

The 6‐week‐old male nude mice used in this assay were purchased from Sippr‐BK Laboratory Animal Co., Ltd. (Shanghai, China). Hep3B cells were transfected with empty plasmids (NC), miR‐552 mimics, *AJAP1* or miR‐552 mimics+*AJAP1* and 1 × 10^6^ cells from each group were digested and resuspended with normal saline and implanted into mice through subcutaneous injections. The observation lasted for 28 days, and the gross tumour volume was measured every 4 days using the formula *V* = 0.5 × *L* × *W* ^2^.

### 2.11 Survival and statistical analysis

The in vitro experiments were performed three times, while the in vivo experiments were performed four times. All data are reported as the mean ± SD. The statistical analysis was performed with SPSS 22.0 using one‐way ANOVA and chi‐squared tests for intergroup comparison. The Pearson correlation coefficient was used for correlation analysis. Prognosis data were downloaded from the TGCA database and analysed (KM analysis) using R project. Differences were considered significant when *P *<* *0.05.

## 3 RESULTS

### 3.1 MiR‐552 was overexpressed in HCC tissues and cell lines

Volcano plot for the selected 42 pairs of HCC and normal tissues with regard to TCGA database were drawn (Figure 1A). As illustrated in the heat map of the 17 most significantly differentially expressed miRNAs shown in Figure 1B, miR‐552 expression was significantly up‐regulated in HCC tissues (*P *<* *0.05). Moreover, the results from RT‐qPCR experiment showed that in comparison with the L02 normal hepatic cells, miR‐552 was found to be notably overexpressed in Hep3B, SMMC‐7721, Bel‐7404 and HepG2 tumour cells (Figure 1C, *P* < 0.05), and its expression was the highest in the Hep3B and HepG2 cells (*P *<* *0.01). Because of their especially high miR‐552 expression level compared with the rest of the cell types, the Hep3B and HepG2 cell lines were chosen for further experiments. It is obvious that miR‐552 was overexpressed in human HCC tissues and cell lines compared with normal counterparts.

### 3.2 MiR‐552 expression level was correlated with clinicopathological characteristics

When the mean value of miR‐552 expression was regarded as a criterion, 35 of the 81 patients belonged to the low‐expression group and 46 belonged to the high‐expression group. A chi‐squared test was used to analyse the correlation between miR‐552 expression level and clinicopathological characteristics of HCC patients. As shown in Table 1, high miR‐552 expression tended to be observed in the higher‐grade category (*P *=* *0.001) and advanced HCC category (*P *=* *0.043). To summarize, miR‐552 level was significantly correlated with tumour histological grade and TNM stage, but was not correlated with the patient's gender, age, AFT level, tumour size and tumour number (all *P *>* *0.05).

### 3.3 MiR‐552 promoted HCC cell proliferation and mobility

**Figure 2:**
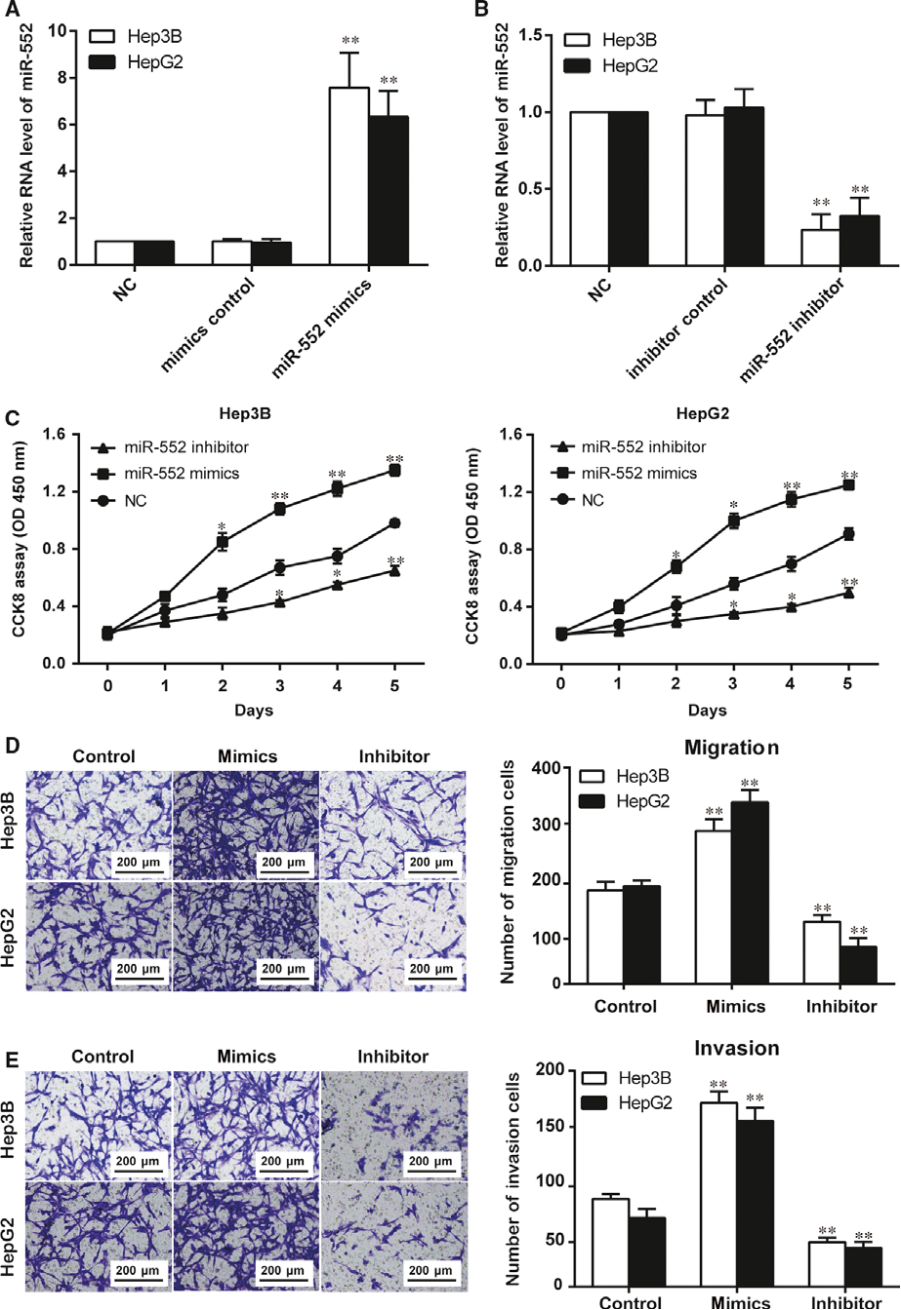
MiR‐552 promoted hepatocellular carcinoma (HCC) cell proliferation, migration and invasion. (A) In both Hep3B and HepG2 cells, miR‐552 was overexpressed in cells transfected with miR‐552 mimics. The cells in the mimic control group showed no significant difference compared with the NC group. **, *P *<* *0.01 compared to the NC group. (B) In both Hep3B and HepG2 cells, miR‐552 was down‐regulated in cells transfected with miR‐552 inhibitors. The cells in the inhibitor control group showed no significant difference compared with the NC group. **, *P *<* *0.01 compared to the NC group. (C) CCK8 assay results indicated that miR‐552 mimics led to a higher absorbance at OD 450 nm while the miR‐552 inhibitor led to a reduced absorbance in both Hep3B and HepG2 cells. *, *P *<* *0.05, **, *P *<* *0.01 compared to the NC group. (D, E) Transwell assay results showed that miR‐552 mimics promoted both migration and invasion in selected HCC cell lines, whereas the miR‐552 inhibitor acted as a suppressor. **, *P *<* *0.01 compared to the control group

We thus suggested that miR‐552 could affect HCC progression. We then conducted in vitro proliferation and mobility experiments. In both the Hep3B and HepG2 cell lines, the increase in miR‐552′s relative RNA level due to transfection with mimics and its decrease due to transfection with inhibitors were marked as significant in comparison with the NC group (Figure 2A,B, *P* < 0.05). The CCK8 assay results demonstrated that miR‐552 upregulation promoted Hep3B and HepG2 cell proliferation, whereas inhibition of miR‐552 expression suppressed proliferation. As illustrated in Figure 2C, the miR‐552 inhibitor group resulted in a lower absorbance than the NC group, while the mimics group had a higher absorbance (*P *<* *0.05). In the Transwell assays, more cells from the miR‐552 mimics group migrated and invaded. Meanwhile, downregulation of miR‐552 limited the two processes (Figure 2D,E, *P* < 0.05). Thus, miR‐552 promotes the proliferation, migration and invasion processes of HCC cells.

### 3.4 MiR‐552 targeted *AJAP1* and inhibited its expression

**Figure 3:**
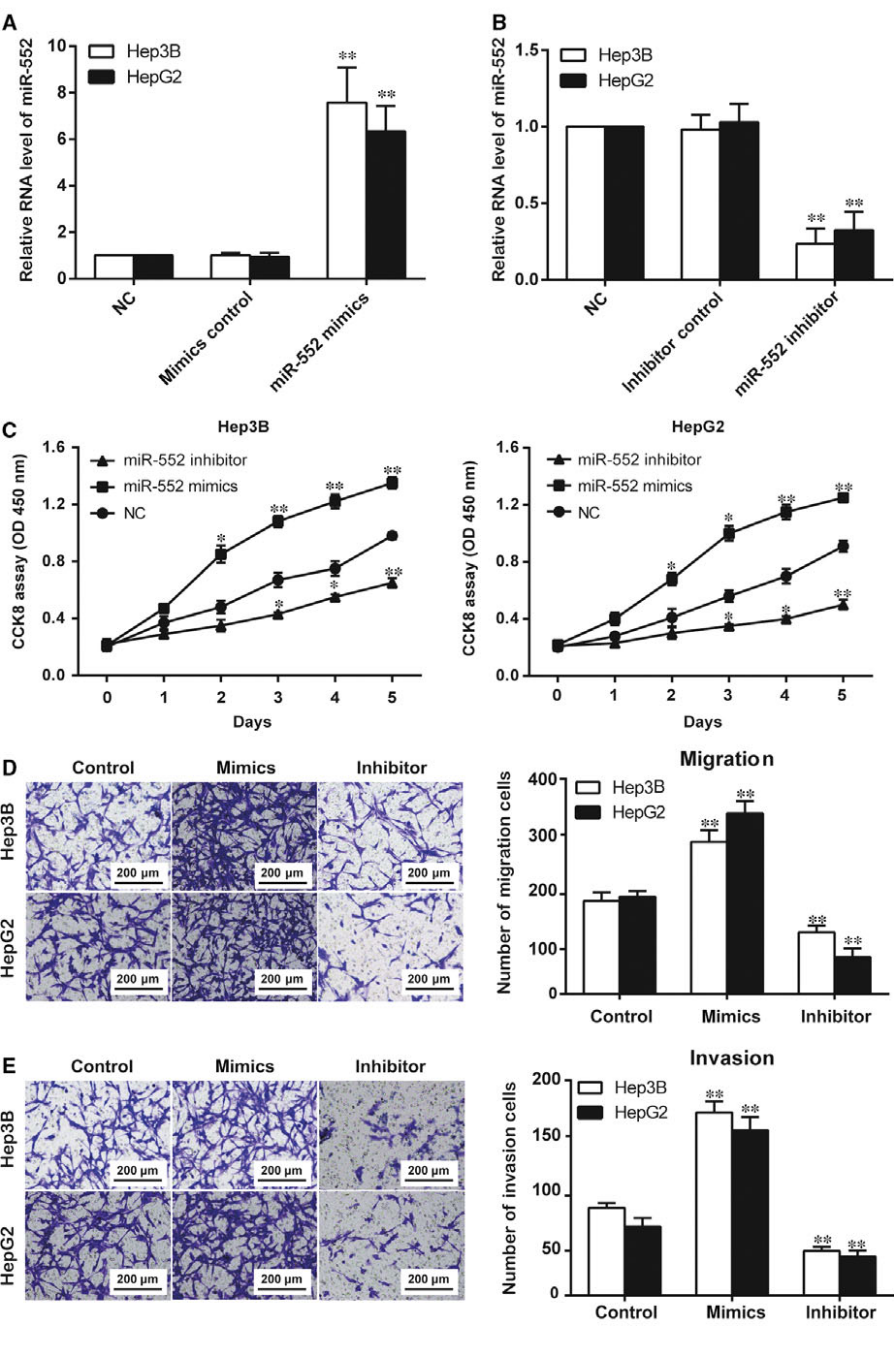
MiR‐552 targeted *AJAP1* and inhibited *AJAP1* expression. (A) The predicted binding sequences of miR‐552 and *AJAP1* were obtained from TagetScanHuman 7.1. The sequences of wild‐type (WT) and mutated (MUT) *AJAP1* are also shown. (B) A luciferase reporter gene assay was used based on the construction of WT *AJAP1* 3’‐UTR and MUT *AJAP1* 3′‐UTR. The relative luciferase activities in the Hep3B and HepG2 cell lines in the WT *AJAP1* 3′‐UTR group were found to be significantly lower than those of cells in the miR‐552 NC group, whereas the MUT *AJAP1* 3′‐UTR group differed insignificantly from its counterpart (the NC group) in both cell lines. *, *P *<* *0.05 compared to the NC group. (C) RT‐qPCR results showed the expression levels of *AJAP1* in Hep3B and HepG2 cells transfected with miR‐552 mimics and miR‐552 inhibitor. *AJAP1* expression was found to be lower when miR‐552 was up‐regulated, yet higher expression was detected in the miR‐552 inhibitor group. *, *P *<* *0.05 compared to the NC group. (D) Western blotting was used to measure the expression level of AJAP1 in Hep3B and HepG2 cells transfected with miR‐552 mimics and miR‐552 inhibitor. The miR‐552 mimics led to lower AJAP1 expression, while the miR‐552 inhibitor led to higher AJAP1 expression. (E) Among the 81 pairs of HCC and adjacent tissues, *AJAP1* was shown to have lower expression in tumour tissues based on the RT‐qPCR experiment. ****, *P *<* *0.0001 compared to the adjacent tissue group. (F) Relative expression of miR‐552 was negatively correlated with that of *AJAP1* in HCC tissues, which was showed a very significant correlation (*P < *0.0001)

A bioinformatics analysis was performed with TagetScanHuman 7.1, which revealed the targeting relationship between miR‐552 and *AJAP1*. The details of the analysis are shown in Figure 3A. Compared with the NC group, both the Hep3B and HepG2 cell lines transfected with WT *AJAP1* 3′‐UTR+miR‐552 mimics had lower relative luciferase activities, while the activities in cells transfected with MUT *AJAP1* 3′‐UTR+miR‐552 mimics were not affected (Figure 3B, *P* < 0.05). We found lower *AJAP1* expression upon miR‐552 overexpression and that miR‐552 knockdown led to increased *AJAP1* expression in the two cell lines based on RT‐qPCR experiments (Figure 3C, *P* < 0.05). Our Western blot outcome agreed with this result (Figure 3D, *P* < 0.05).

The RT‐qPCR results from the 81 pairs of HCC and adjacent tissues showed that *AJAP1* had significantly higher expression in tumour‐adjacent tissues than in HCC tissues (Figure 3E, *P* < 0.0001). In addition, there was a significant inverse correlation between the relative expression levels of miR‐552 and *AJAP1* in HCC tissues (Figure 3F, *P* < 0.0001).

In conclusion, miR‐552 targets *AJAP1* in HCC tissues and cell lines. The expression levels of the two factors are very likely negatively correlated.

### 3.5 MiR‐552 promoted HCC cell proliferation, migration and invasion by inhibiting *AJAP1*

**Figure 4:**
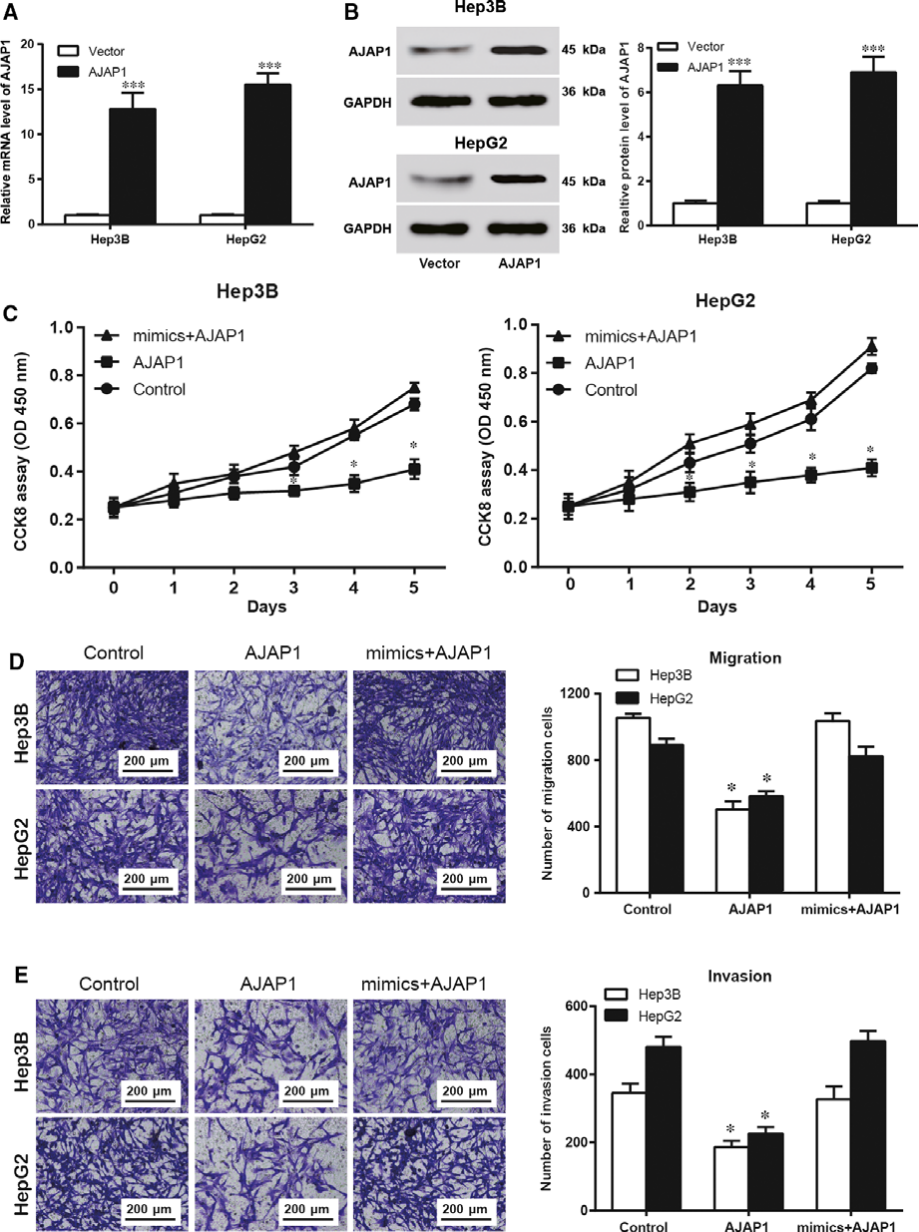
MiR‐552 suppressed hepatocellular carcinoma (HCC) cell proliferation, migration and invasion by targeting *AJAP1*. (A) Hep3B and HepG2 cells transfected with empty vectors or *AJAP1* plasmids were examined using RT‐qPCR. The relative mRNA levels of *AJAP1* were significantly higher in the *AJAP1* groups, indicating successful transfection. ***, *P *<* *0.001 compared to the vector group. (B) The results from Western blot experiment showed higher relative expression levels in cells of the *AJAP1* group than in cells of the vector group for the both Hep3B and HepG2 cell lines. This observation verified successful *AJAP1* transfection. (C) CCK8 assay results showed that in both cell lines, *AJAP1* overexpression suppressed HCC cell proliferation, whereas miR‐552 + *AJAP1* cotransfection had no significant influence on cell proliferation. (D) In the Transwell assay to assess cell migration, fewer cells migrated when *AJAP1* was overexpressed compared with the control group; however, the mimics+*AJAP1* group had almost no difference compared to the control group. (E) In the Transwell assay to assess cell invasion, there was less invasion by AJAP1‐overexpressing cells compared to the control group; however, the mimics+*AJAP1* group had almost no difference. *, *P *<* *0.05 compared to the control group

The results from RT‐qPCR and Western blot experiments showed that the relative mRNA levels and expression levels of *AJAP1* were significantly higher in the *AJAP1* groups, indicating successful transfection (Figure 4A,B, *P* < 0.001). These cells were then used in the CCK8 assay to investigate their proliferative ability. The results showed that in comparison with the control group, overexpression of only *AJAP1* decreased HCC cell proliferation, but that the proliferation rate was not affected by cotransfection with miR‐552 mimics and *AJAP1* plasmids (Figure 4C, *P* < 0.05). Similarly, overexpression of *AJAP1* limited cell migration and invasion, while simultaneous upregulation of miR‐552 and *AJAP1* in HCC cells did not alter cell mobility (Figure 4E, *P* < 0.05). In summary, the promotive role of miR‐552 in HCC cells occurred through inhibition of *AJAP1* expression.

### 3.6 MiR‐552 promoted EMT and oncogenesis of HCC by inhibiting *AJAP1*

**Figure 5:**
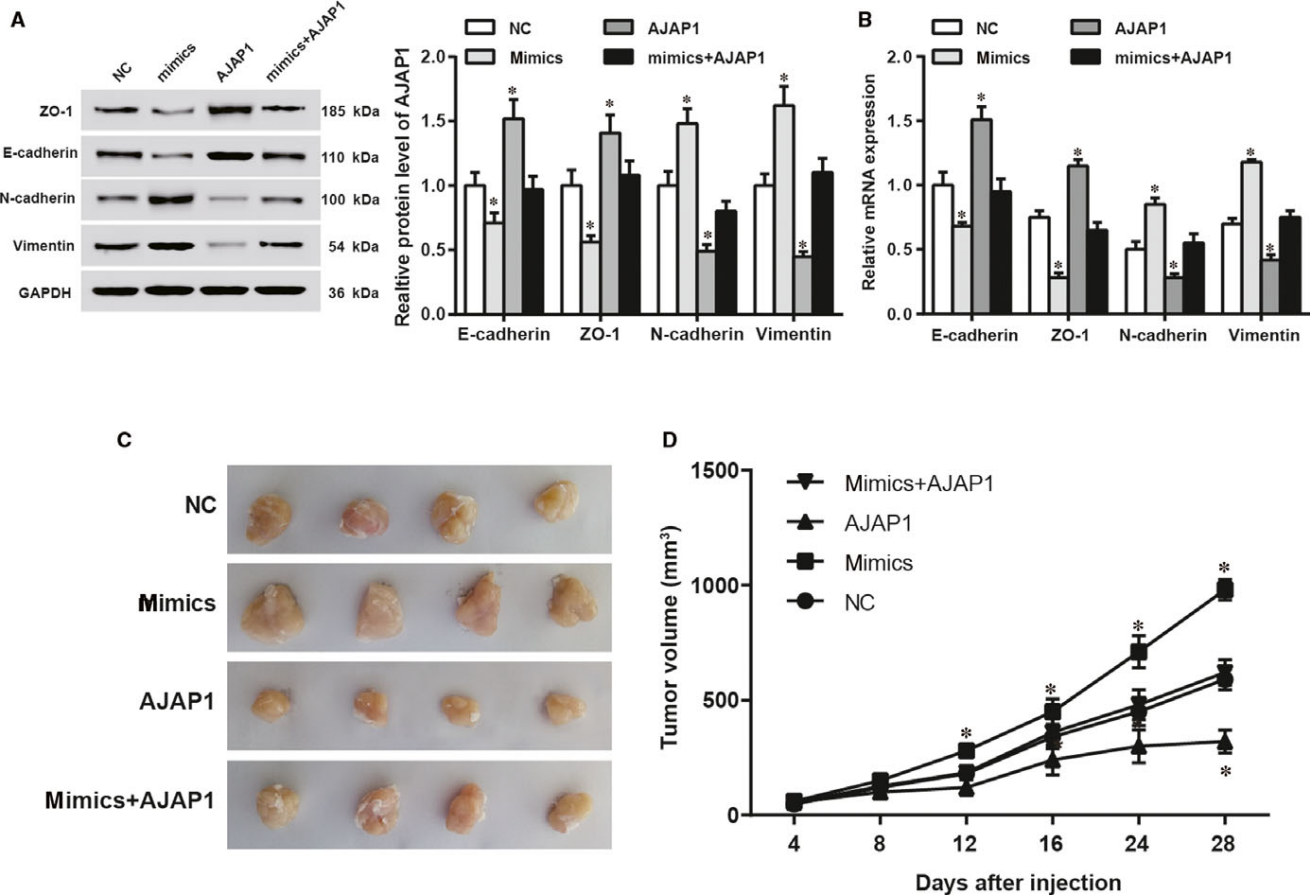
MiR‐552 facilitated EMT and hepatocellular carcinoma (HCC) oncogenesis by downregulating *AJAP1*. (A, B) Using Western blot and RT‐qPCR experiments, we found the inhibitory effects of miR‐552 on the expression levels of E‐cadherin and ZO‐1 and promotive effects on the expression levels of N‐cadherin and Vimentin. *AJAP1* upregulation resulted in higher E‐cadherin and ZO‐1 levels and lower N‐cadherin and Vimentin levels. Overexpression of miR‐552 and *AJAP1* in combination did not influence these expression levels. *, *P *<* *0.05 compared to the NC group. (C, D) A nude mice tumour transplantation assay showed that miR‐552 acted as a tumour promoter and that *AJAP1* acted as an inhibitor in comparison with the NC group. The two in combination did not affect tumour size. *, *P *<* *0.05 compared to the NC group

Hep3B cell lines were used for the upcoming assays. Western blot outcome indicated that in contrast to the NC group, miR‐552 overexpression cut down the relative expression level of E‐cadherin and ZO‐1 and increased the expression levels of N‐cadherin and Vimentin, all of which are EMT marker proteins (*P *<* *0.05). *AJAP1* overexpression elevated the expression levels of E‐cadherin and ZO‐1 and down‐regulated the expression of N‐cadherin and Vimentin (*P *<* *0.05). On the other hand, upregulation of both factors did not influence the expression of the above proteins (Figure 5A, *P* > 0.05). RT‐qPCR results confirmed these results (Figure 5B, *P* < 0.05). A tumour transplantation assay was performed on male mice for in vivo oncogenesis test. Overexpression of miR‐552 promoted tumour growth, but overexpression of *AJAP1* inhibited it. Upregulation of both miR‐552 and *AJAP1* caused no significant change (Figure 5C,D, *P* < 0.05). Based on the above results, we concluded that miR‐552 prompted HCC growth and EMT by regulating *AJAP1*.

### 3.7 Consideration of MiR‐552 and *AJAP1* together provided a better prognosis index for HCC patients

**Figure 6:**
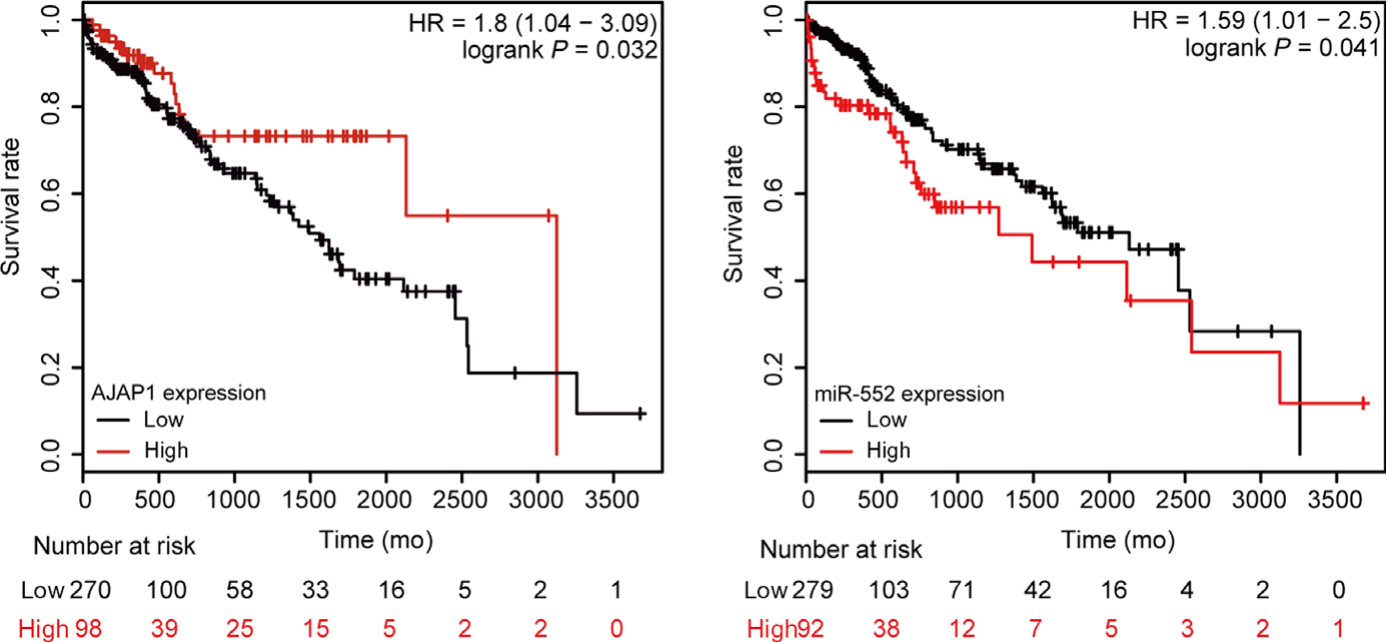
Prognosis results of miR‐552 and *AJAP1* of hepatocellular carcinoma (HCC) patients. (A) The survival rate of HCC patients after complete resection was higher in the high‐*AJAP1*‐level group. *P *<* *0.05 compared to the low‐*AJAP1*‐level group. (B) The survival rate of HCC patients after complete resection was lower in the high‐miR‐552‐level group. *P *<* *0.05 compared to the low‐miR‐552‐level group

The survival outcome of HCC patients (data downloaded from TCGA database) was analysed using KM survival analyses. The results indicated that after resection, HCC patients with a high *AJAP1* expression level and a low miR‐552 expression level had higher survival rates than did those who had a low *AJAP1* expression level and high miR‐552 expression level (Figure 6A,B, *P* < 0.05). These results indicated that miR‐552 and *AJAP1* levels were closely correlated with HCC prognosis.

## 4 DISCUSSION

**Figure 7:**
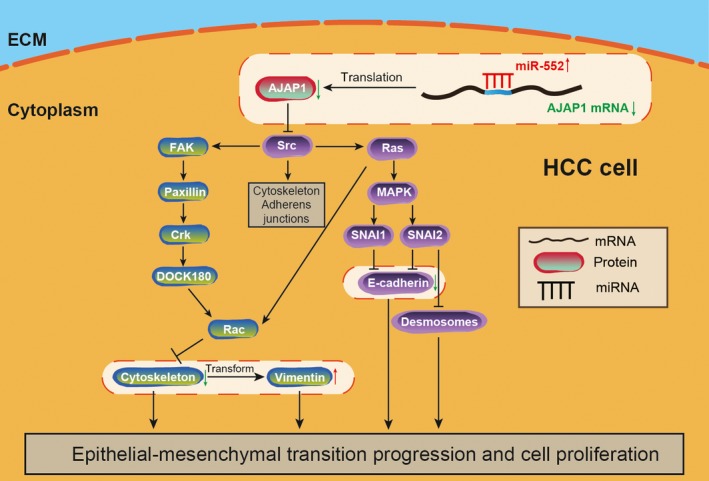
The mechanism of miR‐552 on altering HCC cell behaviours. Overexpression of miR‐522 binds to *AJAP1* mRNA and inhibits its translation. As a result, the level of AJAP1 protein was attenuated, and the level of Src protein, which suppressed by AJAP1, was elevated. The downstream pathway was activated and further promoted EMT progression and cell proliferation. The highlighted elements of the model were verified in our study

In our results, it is obvious that miR‐552 was overexpressed in both the HCC cell lines and in the tissues and was significantly correlated with histological grade and TNM stage. It was also observed that miR‐552 targeted *AJAP1*. By inhibiting *AJAP1*, miR‐552 promoted HCC cell proliferation, migration and invasion in vitro by regulating EMT and stimulated tumour growth in in vivo. Survival analysis indicated higher survival rates in patients with high *AJAP1* expression and low miR‐552 expression than in patients with low *AJAP1* expression and high miR‐552 expression. The potential mechanism of the miR‐522 and *AJAP1* axis is shown in Figure 7. We demonstrated that miR‐552 is a potential HCC biomarker and therapeutic target.

The regulatory roles of miRNA in HCC have long been explored. Sun et al. suggested that miR‐30b suppressed EMT and metastasis in HCC.^19^ The study by Sandbothe et al^20^ revealed the regulatory network of the miR‐449 family in preventing HCC development. In our research, miR‐552 upregulation was confirmed in HCC tissues. Previous studies have shown that miR‐552 is aberrantly expressed along with its regulative function in various human cancers. For example, Kim et al^21^ identified higher miR‐552 expression levels specifically in primary CRC than in lung adenocarcinomas. Xia et al^22^ detected similar upregulation in a subpopulation of colon cancer cells. The promotive role of miR‐552 in CRC was confirmed recently by Wang et al. and Cao et al^6^, ^12^. This paper showed that an understanding of the expression and function of miR‐552 in HCC might help to understand the underlying molecular mechanisms.

We provided evidence to corroborate miR‐552's influence on HCC cell proliferation, migration and invasion as well as its clinical significance. With reference to clinicopathological characteristics, high miR‐552 expression was more likely to appear in patients with higher histological grades or more advanced stages. This finding in HCC partially agrees with reports on CRC by Chen et al^9^, who suggested a significant correlation with TNM stage but not histological grade. However, Kan et al^23^ detected higher miR‐520 g levels in relation to high Edmond‐Steiner grading and more advanced TNM stage, which is largely consistent with our results. Xu et al^1^ observed a correlation between miR‐1296 and TNM stage, but not with HCC grading. Sun et al^24^ argued that there is no correlation between miR‐150 and HCC tumour stage. Our results demonstrated the clinicopathological significance of miR‐552 in HCC, providing a reliable basis for deeper investigations of its role in HCC.

*AJAP1* stood out as the target of miR‐552. By inhibiting *AJAP1* expression, miR‐552 accelerated cell proliferation, migration and invasion. We also found that higher *AJAP1* expression and lower miR‐552 expression levels predicted a higher survival rate. Hötte et al. found that AJAP1 is associated with the cytoskeleton in endothelial cells.^25^ Tanaka et al^26^ pointed out the necessity of exploring the correlation between *AJAP1* expression, a tumour‐recurrence predictor and EMT markers. When miR‐552 was up‐regulated, EMT was activated. Our finding revealed that the underlying molecular mechanism of miR‐552's role in stimulating EMT is via suppression of *AJAP1* in HCC in vitro. In addition, the tumour development in our mouse model supported the in vitro results. As was demonstrated by Han et al^27^, *AJAP1* overexpression in vitro functioned similarly to in vivo overexpression in glioma cell lines. Furthermore, Ezaka et al^16^ proved that *AJAP1* levels were inversely correlated with the levels of *SRC* in HCC cell lines and tissues, which verified our hypothesis shown in Figure 7. We also demonstrated effects on the downstream proteins E‐cadherin and Vimentin, which also support the effects of miR‐552 and *AJAP1* on EMT in HCC. Therefore, we concluded that miR‐552 could facilitate HCC by promoting the EMT pathway.

There are still limitations in the current paper. For instance, a previous study proved that transfection of miRNA mimics at high concentrations altered the gene expression in a nonspecific manner and can cause cell death,^28^ suggesting that the results may be affected by artefacts. The transfections used in our research were all transient transfection. As it is efficient in vitro, lentiviral transfection is more valid in vivo. These methods still need to be improved. On the other hand, metastasis assays could also be used in further studies to explore whether miR‐552 could influence HCC metastasis. In vivo experiments on the inhibition of distant metastases will be performed in future studies. Because HCC is highly metastatic, further study of HCC metastasis would increase the clinical significance of using miR‐552 as a therapy target for its treatment. Furthermore, we only proved a part of our hypothesis, and further study will be performed in the future.

MiR‐552 was expressed at a high level in HCC tissues and cell lines. Upregulation of miR‐552 was positively correlated with HCC cell proliferation and migration, along with poor clinicopathological characteristic of postoperative patients. MiR‐552 played an oncogenic role by restricting *AJAP1* expression and manipulating EMT‐related protein levels. Higher miR‐552 and lower *AJAP1* levels also corresponded to poorer HCC prognoses. In conclusion, miR‐552 induced HCC progression by downregulating *AJAP1* and may be significant for future HCC treatment.

## ETHICS APPROVAL

The study was approved by the Ethics Boards of Qingdao No. 6 People's Hospital.

## CONSENT FOR PUBLICATION

Written informed consent was collected from all patients.

## CONFLICT OF INTERESTS

The authors declare that they have no competing interests.

## AUTHORS’ CONTRIBUTIONS

Contributing to the conception and design: Keli Su, Weiqing Qu; Analysing and interpreting data: Xinyuan Wen; Drafting the article: Weiqing Qu and Xinyuan Wen; Revising it critically for important intellectual content: Wei Gou; Approving the final version to be published: All authors.
